# Effectiveness of non-invasive chromosomal screening for normal karyotype and chromosomal rearrangements

**DOI:** 10.3389/fgene.2023.1036467

**Published:** 2023-03-13

**Authors:** Bo-lan Sun, Yong Wang, Sixi-Wen, Liang Zhou, Chun-hui Zhang, Ze-Xuan Wu, Jie Qiao, Qing-yuan Sun, Ya-xin Yao, Jing Wang, Zi-Yun Yi, Wei-Ping Qian

**Affiliations:** ^1^ The Reproductive Medicine Center, Peking University Shenzhen Hospital, Shenzhen, Guangdong, China; ^2^ Reproductive Medical Center, Peking University Third Hospital, Beijing, China; ^3^ Fertility Preservation Lab, Reproductive Medicine Center, Guangdong Second Provincial General Hospital, Guangzhou, China; ^4^ Department of Clinical Research, Yikon Genomics Company, Ltd., Suzhou, China

**Keywords:** non-invasive chromosomal screening, assisted reproductive technology, chromosomal ploidy, next-generation sequencing, blastocyst culture medium, clinical outcomes

## Abstract

**Purpose:**

To study the accuracy of non-invasive chromosomal screening (NICS) results, in normal chromosomes and chromosomal rearrangement groups and to investigate whether using trophoblast cell biopsy along with NICS, to choose embryos for transfer can improve the clinical outcomes of assisted pregnancy.

**Methods:**

We retrospectively analyzed 101 couples who underwent preimplantation genetic testing at our center from January 2019 to June 2021 and collected 492 blastocysts for trophocyte (TE) biopsy. D3-5 blastocyst culture fluid and blastocyst cavity fluid were collected for the NICS. Amongst them, 278 blastocysts (58 couples) and 214 blastocysts (43 couples) were included in the normal chromosomes and chromosomal rearrangement groups, respectively. Couples undergoing embryo transfer were divided into group A, in which both the NICS and TE biopsy results were euploid (52 embryos), and group B, in which the TE biopsy results were euploid and the NICS results were aneuploid (33 embryos).

**Results:**

In the normal karyotype group, concordance for embryo ploidy was 78.1%, sensitivity was 94.9%, specificity was 51.4%, the positive predictive value (PPV) was 75.7%, and the negative predictive value (NPV) was 86.4%. In the chromosomal rearrangement group, concordance for embryo ploidy was 73.1%, sensitivity was 93.3%, specificity was 53.3%, the PPV was 66.3%, and the NPV was 89%. In euploid TE/euploid NICS group, 52 embryos were transferred; the clinical pregnancy rate was 71.2%, miscarriage rate was 5.4%, and ongoing pregnancy rate was 67.3%. In euploid TE/aneuploid NICS group, 33 embryos were transferred; the clinic pregnancy rate was 54.5%, miscarriage rate was 5.6%, and ongoingpregnancy rate was 51.5%. The clinical pregnancy and ongoing pregnancy rates were higher in the TE and NICS euploid group.

**Conclusion:**

NICS was similarly effective in assessing both normal and abnormal populations. Identification of euploidy and aneuploidy alone may lead to the wastage of embryos due to high false positives. More suitable reporting methods for NICS and countermeasures for a high number of false positives in NICS are needed. In summary, our results suggest that combining biopsy and NICS results could improve the outcomes of assisted pregnancy.

## 1 Introduction

With the increase in work pressure and competition in modern society, the average childbearing age of couples is increasing; there is also a decline in fertility rates and an increase in infertility due to various reasons, such as chromosomal abnormalities of embryos which is mainly caused by age-dependent chromosome segregation errors during meiosis I (Xu et al., 2016). Therefore, an increasing number of couples are getting pregnant through assisted reproductive technology. However, chromosomal aneuploidy is an important factor that affects the success rate of assisted pregnancies. Chromosomal abnormalities can be prevented by performing embryo biopsies and preimplantation genetic testing (PGT). Multiple clinical trials have confirmed the clinical efficacy of PGT, including increased rates of implantation and clinical pregnancy, and decreased rates of miscarriages (Dreesen et al., 2014). However, biopsy of blastocyst trophoblast cells that are commonly used in clinics for PGT analysis is an invasive detection method and there are limitations to its clinical application: the biopsy is difficult to carry out and damage to embryos cannot completely be avoided (Zhang J. et al., 2019; Tiegs et al., 2019; Tocci, 2020; Makhijani et al., 2021). Furthermore, 30%–40% of embryos have chromosomal karyotype chimerism, and trophocyte (TE) biopsies cannot accurately represent the genome profile of the inner cell mass (ICM) and the remainder of the TE. Therefore, the accuracy of PGT detection is reduced by sample bias (Taylor et al., 2014; Munné et al., 2020).

Given the above limitations of TE biopsies, recent attention has been given to a non-invasive chromosome detection approach in embryos. Both genomic and mitochondrial DNA contents were found in blastocoel fluid (also called blastocyst cavity fluid; BF) and spent culture medium (Magli et al., 2016). In 2013, Stigliani et al. (Stigliani et al., 2013) first confirmed the existence of cell-free DNA in blastocyst culture media (SCM). Subsequently, DNA in the BF could be used for genetic analysis (Basile et al., 2015; Chen L. et al., 2021). Xu et al. reported a non-invasive chromosomal screening (NICS) method based on the sequencing of genomic DNA secreted into the culture medium from human blastocysts. This approach has the potential for a much wider chromosome screening applicability in clinical *in vitro* fertilization, due to its high accuracy and non-invasiveness (Gianaroli et al., 2014; Xu et al., 2016; Leaver and Wells, 2020; Rubio et al., 2021). Previous studies have shown that DNA testing using an embryo culture medium could on days 5 or 6 detect chromosome abnormalities with a reasonable positive predictive value (PPV) and high negative predictive value (NPV) (Rubio et al., 2021). Moreover, NICS, which is based on the sequencing of DNA from the SCM, may better represent the entire embryo compared to a TE biopsy alone (Poli et al., 2019). Moreover, in another study, concordance was higher in the SCM with the BF DNA analysis combination than in PGT with the TE biopsy alone (Huang et al., 2019). The one limitation to these methods is the low amount of DNA present in the BF and SCM; this can now be amplified for genetic analysis through whole-genome amplification (WGA) and detected through array comparative genomic hybridization and next-generation sequencing (NGS) (Shamonki et al., 2016; Jiao et al., 2019). In addition to this, several important issues need to be addressed before the routine clinical application of NICS. These include minimization of maternal DNA contamination (Kuznyetsov et al., 2018), determining factors that affect accuracy, and optimization of the WGA protocol for DNA in the SCM and BF.

For embryo screening, both the trophoblast cell biopsy and NICS methods have their advantages and disadvantages. We wanted to understand whether combining the two methods, to select embryos for transfer, improves the clinical outcome, as there are few such reports.

Therefore, in this study, we compared the consistency, sensitivity, specificity, PPV, and NPV of NICS in normal chromosome and chromosomal rearrangement groups. This is the first time that such a large sample size of different populations has been used to evaluate the performance of NICS. Furthermore, based on the results of TE biopsies and NICS, patients undergoing an embryo transfer were divided into two groups: euploid TE/euploid NICS group in which both the TE biopsy and NICS results suggested euploidy, and euploid TE/aneuploid NICS group in which the TE biopsy results suggested euploidy and the NICS results suggested aneuploidy. A series of clinical results from the two groups were compared in order to determine whether TE biopsies combined with NICS could improve clinical outcomes.

## 2 Materials and methods

### 2.1 Study population

We performed a retrospective analysis of 101 couples who received PGT in our center from January 2019 to June 2021; 492 blastocysts were collected for TE biopsy. Meanwhile, D3-5 SCM and BF were collected for NICS. Among them, 278 blastocysts (58 couples) were included in the normal chromosome group and 214 blastocysts (43 couples) were included in the chromosomal rearrangement group. This study was approved by the Ethics Committee of the Peking University of Shenzhen Hospital ([2018] Issue no [014]) and was performed in accordance with the principles of the Declaration of Helsinki (1964) and its later amendments. Informed consent was obtained from all the patients included in this study.

### 2.2 SCM, BF collection, and TE biopsy

In our study, we carefully removed and washed the cumulus-corona colliculus complex before intracytoplasmic sperm injection (ICSI). The embryos derived from ICSI were cultured to cleavage stage (D3). If there were any remaining granulosa cells, we removed the ramaining granulosa cells completely with a glass pipe. Then, each embryos was rinsed and transferred into an 20 µL droplet of blastocyst media, in the BD353001 petri dish (BD medical, Franklin Lakes, United States) to equilibration overnight. On the afternoon of D4, the embryos cultured in the collective culture were repeatedly blown, washed, and placed successively into the previously prepared blastocyst culture medium. Each embryo was placed into an individual drop of fresh blastocyst culture medium, which was then cultured in a three-gas incubator (37°C, 6% CO_2_, 5% O_2_). After the D5-D6 balstocyst was completely formed, artificial collapse of the blastocoel was induced by applying a laser pulse (300 μs), using the ZILOS-tkTM laser system (Hamilton Thorn Bioscience Inc., Beverly, MA, United States), at the junction of TE cells and the location of the juction was far from the inner cell mass. After 5 min of treatment, all the cluture media with the released blastocyst fluid were collected with a drawn glass straw and placed into a PCR tube containing 5 µL of cell lysis buffer (Yikon Genomics, Shanghai, China). The shriveled blastocyst was put into the biopsy operation dish and biopsied under an inverted microscope. The fixed needle was used to hold the cell mass inside the blastocyst at 9 o’clock, and a small hole was punctured, in the trophoblast cell junction, at three points opposite to the ICM. The biopsy needle entered the blastocyst at the perforated position and absorbed 3–6 trophectoderm ectoderm cells. The biopsied cells were completely separated from the blastocyst by a laser pulse (300 μs), using the ZILOS-tkTM laser system (Hamilton Thorn Bioscience Inc., Beverly, MA, United States), while being pulled. The biopsied blastocyst was removed under a stereomicroscope, and the biopsied trophoblast ectoderm cells were cleaned several times in phosphate-buffered saline. The egg stripping pipette (135 µm inner diameter) was used to move the biopsied trophoblast ectoderm cells into the PCR tube containing 5 µL of cell lysis buffer.

### 2.3 WGA and library preparation for NGS

The SCM, BF and TE biopsy samples were subjected to cell lysis followed by WGA with the multiple annealing and looping-based amplification cycles (MALBAC) technique and library generation as described previously. The amplification products were sequenced on the Illumina HiSeq 2,500 platform (Illumina, San Diego, CA, United States) with approximately two million sequencing reads per sample. The read numbers were counted along the whole genome with a bin size of 1 Mb and normalized based on GC content and a reference dataset. A copy number gain from two to three copies results in a 50% increase in read counts, whereas a copy number loss from two copies to one copy results in a 50% decrease in read counts.

The standard for mosaicism in NICS (Jiao et al., 2019; Rubio et al., 2019; Yeung et al., 2019) and TE-PGT (Cram et al., 2019; Yeung et al., 2019; Lin et al., 2020) is not yet unified. We use the 30% as the threshold of TE-PGT. In the study reported by Yeung et al., the threshold of NICS and TE-PGT was also 30% (Yeung et al., 2019). For the detection results of TE cells and NICS, the mosaicism was reported as 30%. Embryos were classified as “mosaicism” if their mosaicism ranged from 30% to 70%. Embryos were classified as “euploid” if they were less than 30% mosaicism. Embryos were classified as “aneuploid” if they were more than 70% mosaicism. Due to the small fragment size and very minute DNA concentration, the window size for analysis was larger because sequencing date for SCM and BF were relatively noisier than for TE; and therefore, the detection limit was down to sub-chromosomal level (Yeung et al., 2019). Here, For the detection results of TE cells and SCM and BF results, the abnormal fragments were reported as ≥4 Mb and ≥10 Mb.

**FIGURE 1:**
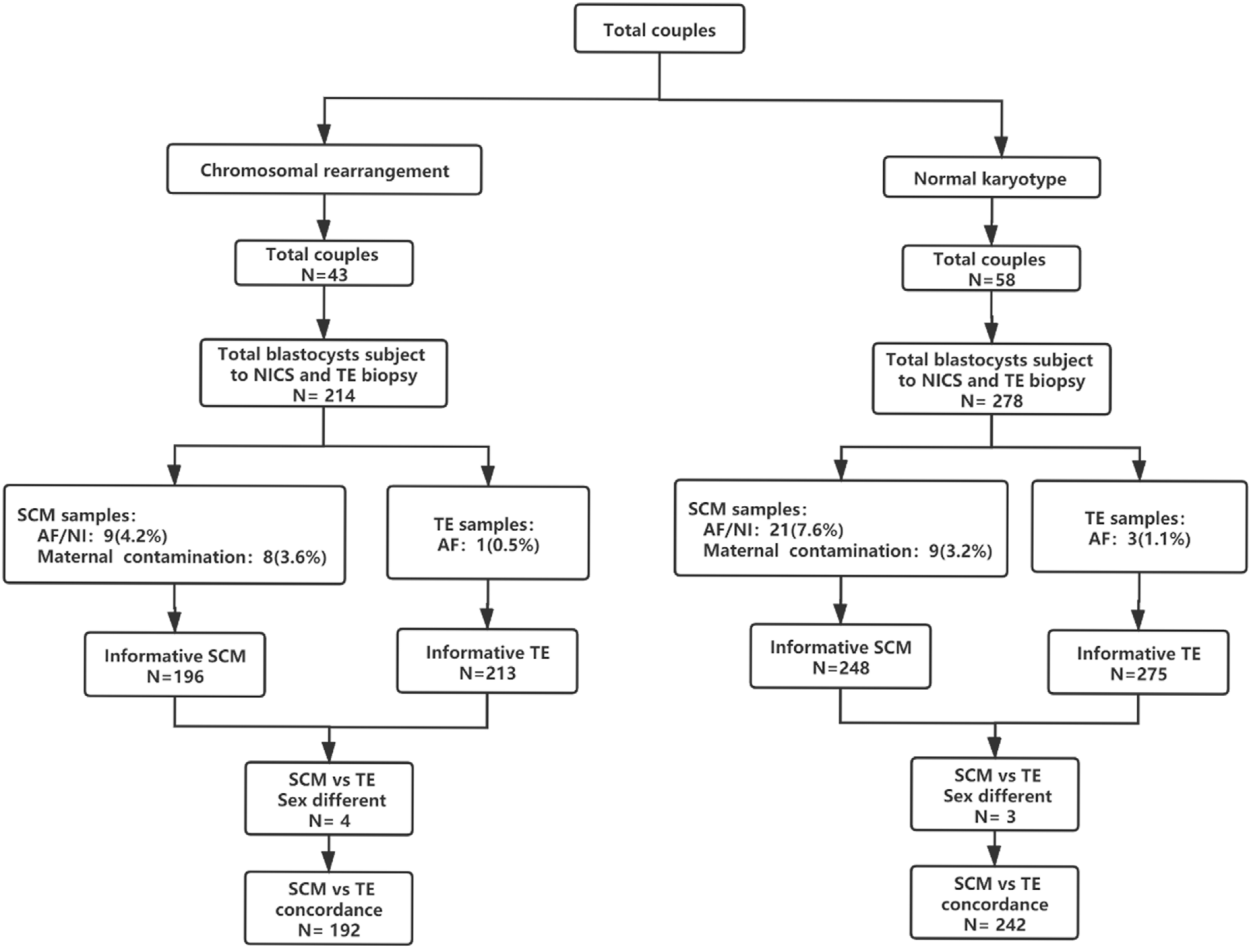
Flowchart of the study design. A total of 101 couples were included in this study; 278 blastocysts (58 couples) were included in the normal chromosome group and 214 blastocysts (43 couples) were included in the chromosomal rearrangement group. Abbreviations: AF, amplify failure; NI, non-information; NICS, non-invasive chromosomal screening; SCM, blastocyst culture fluid; TE, trophocyte.

**FIGURE 2:**
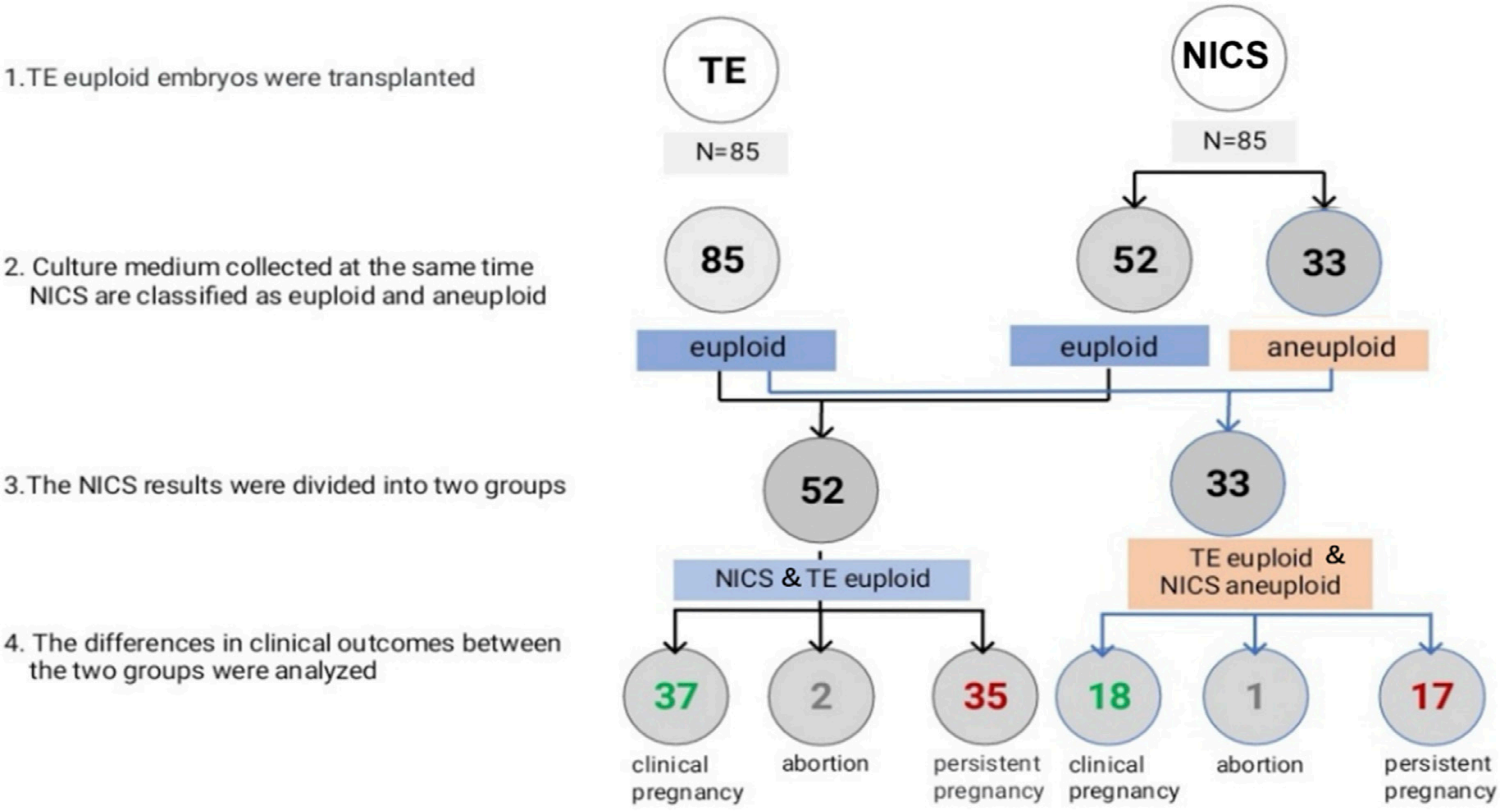
Euploidy and clinical outcomes of the transferred embryos. Abbreviations: NICS, non-invasive chromosomal screening; TE, trophocyte.

First, the NICS results were compared to the TE biopsy results in both groups. The consistency, sensitivity, specificity, PPV, and NPV were calculated. The parameters between the two groups were statistically analyzed (Figure 1). Thereafter, according to the TE biopsy and NICS results, couples undergoing embryo transfer were divided into two groups: euploid TE/euploid NICS group, wherein both the TE biopsy and NICS showed euploidy (52 embryos) and euploid TE/aneuploid NICS group, wherein the biopsy results showed euploidy and the NICS results showed aneuploidy (33 embryos). The clinical results from both groups (clinical pregnancy rate, miscarriage rate, ongoing pregnancy rate) were compared (Figure 2). In terms of outcomes, clinical pregnancy was diagnosed when a gestational sac was ascertained by transvaginal ultrasonography, ongoing pregnancy was defined as continued pregnancy at 8–12 weeks with positive fetal cardiac activity, while clinical miscarriage was defined as loss of pregnancy after some ultrasound findings were present (at least a gestational sac).

### 2.4 Statistical analysis

Descriptive data for the continuous variables are presented as the mean and standard deviation, and categorical data as numbers and percentages. Student’s *t*-tests and Mann–Whitney tests were used for parametric and non-parametric data, respectively. The chi-square or Fisher exact tests were used for the categorical variables of each group. All analyses were done using the SPSS version 25.0 (IBM, Armonk, NY, United States). Multiple logistic regression analyses were conducted to compare the outcomes of group A and group B after controlling for *p* < 0.10 and co-variables considered clinically influential, including female age, morphology (expansion, ICM, and trophectoderm), and cycle type of embryo transfer.

## 3 Results

There were 278 blastocysts in the normal karyotype group; amongst these 9 blastocysts were contaminated with the parent source, 3 blastocysts were contaminated by exogenous sources (the extenrnal DNA may derived from plasticware, media, or manipulation during IVF), 21 blastocysts DNA samples from SCM and BF failed to amplify and could not be detected by NICS, and 3 blastocysts failed to amplify and could not be detected by TE biopsy. Finally, 242 blastocysts were used for consistent comparison (Figure 1). In the blastocyst culture medium of the 242 blastocysts included in the final analysis, the consistency rate was 73.1%, sensitivity was 93.3%, and specificity was 53.3%. There were 65 true negatives and 8 false negatives, with a false negative rate of 6.7% and an NPV of 89%. There were 112 true positives and 57 false positives, with a false positive rate of 46.7% and a PPV of 66.3% (Table 1).

**TABLE 1:** Comparison of various parameters between the normal chromosome group and rearrangement group.

Performance characteristic	Chromosomal rearrangement group (*n* = 192)	Normal karyotype group (*n* = 242)
FPR (FP/TN + FP)	48.6% (36/74)	46.7% (57/122)
FNR (FN/TP + FN)	5.1% (6/118)	6.7% (8/120)
Sensitivity (TP/TP + FN)	94.9% (112/118)	93.3% (112/120)
Specificity (TN/TN + FP)	51.4% (38/74)	53.3% (65/122)
PPV (TP/TP + FP)	75.7% (112/148)	66.3% (112/169)
NPV (TN/TN + FN)	86.4% (38/44)	89.0% (65/73)
% Concordance for embryo ploidy	78.1% (150/192)	73.1% (177/242)

Abbreviations: FN, false negative; FNR, false negative rate; FP, false positive; FPR, false positive rate; NPV, negative predictive value; PPV, positive predictive value; TN, true negative; TP, true positive.

There were 214 blastocysts in the chromosomal rearrangement group; amongst these 8 blastocysts were contaminated with the parent source, 4 blastocysts were contaminated by exogenous sources, 9 blastocysts DNA samples from SCM and BF failed to amplify and could not be detected by NICS, and 1 blastocyst failed to amplify and could not be detected by TE biopsy. Finally, 192 were used for consistent comparison (Figure 1). In the blastocyst culture medium of the 192 blastocysts included in the final analysis, the consistency rate was 78.1%, sensitivity was 94.9%, and specificity was 51.4%. There were 38 true negatives and 6 false negatives, with a false negative rate of 5.1% and an NPV of 86.4%. There were 112 true positives and 36 false positives, with a false positive rate of 48.6% and a PPV of 75.7% (Table 1). There was no statistically significant difference (*p* > 0.05) in the performance characteristics between the chromosomal rearrangement and normal karyotype groups.

We further evaluated the embryos with different NICS CNV results. According the classification method of previous research (Xie et al., 2022), The CNV results from BCM can be classified into four different categories: 1) euploidy, 2) low-level mosaics (mosaic rates ≤50%) 3) high-level mosaics (mosaic rates >50%) 4) aneuploidy (Table 2). There was no statistically significant difference (*p* > 0.05) in the performance characteristics between the chromosomal rearrangement and normal karyotype groups.

**TABLE 2:** Comparison of different NICS results between the normal chromosome group and rearrangement group.

NICS	Chromosomal rearrangement group (*n* = 192)	Normal karyotype group (*n* = 242)
Euploidy	22.9% (44/192)	30.2% (73/242)
Low-level mosaics (mosaic rates≤50%)	15.6% (30/192)	19.8% (48/242)
High-level mosaics (mosaic rates>50%)	15.1% (29/192)	12.8% (31/242)
Aneuploidy	46.4% (89/192)	37.2% (90/242)

In all, 85 TE euploid embryos were transferred, which were further divided into two groups: euploid TE/euploid NICS (52 embryos) and euploid TE/aneuploid NICS (33 embryos) groups (Figure 2). There was no statistical difference in any other basic conditions between the two groups except the endometrial preparation protocol (Table 3). The clinical pregnancy rates of the euploid TE/euploid NICS group and the euploid TE/aneuploid NICS group were 71.2% and 54.5%, ongoing pregnancy rates were 69.2% and 51.5%, and miscarriage rates were 5.4% and 5.6%, respectively. There were no statistical differences observed in terms of clinical pregnancy, miscarriage, and ongoing pregnancy rates between the two groups, however, the clinical pregnancy and ongoing pregnancy rates were higher in the euploid TE/euploid NICS group (Table 4).

**TABLE 3:** Clinical baseline characteristics of patients.

	NICS aneuploid	NICS euploid	Overall	*p*-value
Number of patients	31	51	82	
Female age (years)	34.82 ± 4.98	33.79 ± 4.46	34.19 ± 4.67	0.324
Female body mass index	21.39 ± 1.63	21.16 ± 2.62	21.25 ± 2.28	0.621
Infertility duration (years)	2.18 ± 1.57	2.40 ± 1.99	2.32 ± 1.83	0.589
Number of prior miscarriages				
0	57.6%	50%	52.9%	0.495
1∼2	30.3%	38.5%	35.3%	0.443
≥3	12.1%	11.5%	11.8%	1
Number of previous transferred cycles				
0	72.7%	75%	74.1%	0.816
1∼2	18.2%	17.3%	17.6%	0.918
≥3	9.1%	7.7%	8.2%	1
Type of infertility				
Primary	39.4%	36.5%	37.6%	0.791
Secondary	60.6%	63.5%	62.4%	0.791
Number of prior live births	15.2%	17.3%	16.5%	0.794
Number of embryos transferred	33	52	85	
Endometrial preparation protocol				
Natural cycle	9.1% (3/33)	15.4% (8/52)	12.9% (11/85)	0.609
Hormone replacement therapy	75.8% (25/33)	40/4% (21/52)	54.1% (46/85)	**0.001**
Ovulation-inducing cycle	15.2% (5/33)	44.2% (23/52)	32.9% (28/85)	**0.005**
Endometrial thickness (mm)	10.80 ± 2.25	10.70 ± 2.45	10.74 ± 2.36	0.862
Hormone level				
AMH (ng/mL)	4.43 ± 2.26	4.29 ± 2.32	4.34 ± 2.28	0.78
FSH (mIU/mL)	7.58 ± 2.94	8.26 ± 3.27	7.99 ± 3.14	0.332
LH (mIU/mL)	4.47 ± 1.64	4.63 ± 2.54	4.57 ± 2.23	0.738
E2 (pg/mL)	40.21 ± 17.60	48.67 ± 65.18	45.34 ± 51.93	0.47
Embryo Morphology				
Good (AA/BA/AB/BB)	66.7% (22/33)	69.2% (36/52)	68.2% (58/85)	0.805
Poor (AC/CA/BC/CB)	33.3% (11/33)	30.8% (16/52)	31.8% (27/85)	

Abbreviations: AMH, anti-Müllerian hormone; E2, estradiol; FSH, follicle-stimulating hormone; LH, luteinizing hormone; NICS, non-invasive chromosomal screening.

The bold values: There was a significant difference between the euploid TE/euploid NICS and the euploid TE/aneuploid NICS groups, *p* < 0.05.

**TABLE 4:** Comparison of clinical outcomes between the euploid and aneuploid non-invasive chromosomal screening groups in trophocyte euploid embryo transplantation.

	NICS euploid	NICS aneuploid	Unadjusted *p*-value	^a^Adjusted *p*-value	^a^Adjusted odds ratio (95% CI)	Total
Transferred euploid blastocysts	52	33				85
Clinical pregnancies	71.2 (38/52)	54.5% (18/33)	0.079	0.095	2.27 (0.87–5.92)	65.9% (56/85)
Miscarriages	5.3% (2/38)	5.6% (1/18)	1	0.975	0.96 (0.06–15.36)	5.4% (3/56)
Ongoing pregnancy	69.2% (36/52)	51.5% (17/33)	0.1	0.123	2.13 (0.82–6.78)	62.4% (53/85)

^a^ Adjusted for age, embryo morphology, and cycle of embryo transfer.

Abbreviations: CI, confidence interval; NICS, non-invasive chromosomal screening.

## 4 Discussion

In recent years, more and more scholars have studied the effectiveness of NICS. Although NICS has the advantage of being a relatively simple and non-invasive sampling process compared to TE biopsy, contamination from the mother source and the low content of DNA in the culture medium can easily lead to the failure of amplification and affect the results. Wei Qiang Liu et al., observed that the DNA dectiction rate in SCM was slightly higher than that in BF samples (Liu et al., 2017), a finding that was also reported by Magil et al. (76.5%) and the Gianaroli group (82%) (Gianaroli et al., 2014) (Magli et al., 2016). Valeriy Kuznyetsov et al. (Kuznyetsov et al., 2018) and Li et al. (Li et al., 2018) found the higher DNA amplification rates were due to increased DNA availability caused by mixing BF and SBM. Subsequently, we collected samples after collapsingg the blastocyst by a laser without causing any other harm to the embryo. The BF DNA can be released into the embryo culture medium, which could notably increases the concerntration of the cf DNA. The rate of successful amplification across BCM samples was 93.9% (462 of 492), and the successful amplification occurered in 99.19% (488 of 492) of TE samples. Since the blastocyst was shriveled with a laser during collection, the protocal could be called less invasive and may be more appropriate than non-invasive. However, in the clinic, before blastocyst vitrification, people, release the blastocoel fluid through laser drilling so that to decrease the formation of ice crystals, which may affect the survival of the blastocyst (Zhang W. Y. et al., 2019). Mukaida et al., reported that the artificial shrinkage of blastocoels by microneedle or alaser pulse before vitrification improves the survival rate and clinical outcome of the embryo (Mukaida et al., 2006).

Maternal contamination is a common problem among discordant results. It is known that contamination by maternal DNA often leads to female bias in the sex ratio. Our research found that 17 samples had gender inconsistency, the TE sex chromosome was XY and the BCM was XX, which may be due to maternal contamination. Our maternal contamination rate (17 of 492) is lower than previously reported: 2 out of 27 (Zhang J. et al., 2019), and higher that of Jiao et al.’ research (0 of 41) (2019). Prevention is very critical. In our study, we carefully removed and washed the cumulus-corona colliculus complex before ICSI and again on the afternoon of day 4. And we wash and replace the culture medium on the afternoon of day 4. We have observed 7 male NICS results coming from a TE-diagnosed female embryo, indicating that external DNA contamination resulting from plasticware, media, or manipulation during IVF is crucial, and caution should be taken to prevent this. Human serum albumin in the embryo culture medium contains human DNA. The content of human DNA in different embryo culture medium batches was slightly different, and a high human DNA content may affect the sequencing result. Decreasing the volume of embryo culture medium or using serum free medium may be helpful for this issue (Zhang W. Y. et al., 2019). Here we use small volume of medium to culture the embryos (20 μL).

In previous studies, the consistency rate of NICS was low (Wong et al., 2014; Magli et al., 2016; Li et al., 2018; Vera-Rodriguez et al., 2018; Huang et al., 2019; Rubio et al., 2020; Chen Y. et al., 2021). Currently, there have been no studies done on the effectiveness of NICS, specifically for people with chromosomal abnormalities. We have compared the consistency, sensitivity, specificity, PPV, and NPV of NICS in the normal chromosome and chromosomal rearrangement groups. The consistency rates were 73.1% and 78.1% in the normal chromosome and chromosomal rearrangement groups, respectively, and did not show any statistical difference. There were also no significant differences in any other characteristics between the two groups. This suggested that NICS was equally effective in both the normal chromosome and chromosomal rearrangement groups. In our study, the sensitivity of the normal chromosome and chromosomal rearrangement groups was 93.3% and 94.9%, specificity was 53.3% and 51.4%, PPV was 66.3% and 75.7%, and NPV was 89% and 86.4%, respectively. Recently, Chen et al. used a whole embryo as the gold standard for conducting a large sample size study on the accuracy of NICS (Chen L. et al., 2021); the sensitivity (87.36%), PPV (73.08%), and NPV (91.2%) are consistent with our findings. A higher NPV indicates higher accuracy in determining NICS as euploidy, a poor PPV indicates poor accuracy in determining NICS as aneuploidy, and that there are high false positives. Mosaicism affects 30%–40% of human blastocyst (Huang et al., 2019). The TE test results may fail to reflect the genome profile of the inner cell mass (ICM), which ultimately forms the fetus (Huang et al., 2019). While the DNA in SCM likely originates form both of the ICM and TE cells, as they both undergo apoptosis during preimplantation development (Huang et al., 2019; Jiao et al., 2019). And, some embryos had different karyotypes in TE *versus* whole embryos and NICS, mostly because of mosaics (Jiao et al., 2019). What’s more, during embryonic development, some abnormal cells die and their DNA is released into the culture medium, which is the process of embryo self-repair (Vera-Rodriguez and Rubio, 2017; Huang et al., 2019). This self-repair process may lead to an increase in the false positive rates of the SCM. The above explanation might explain why some embryos were found to be aneuploid with the non-invasive method and euploid with the TE biopsy. Identification of euploidy and aneuploidy alone may lead to the wastage of embryos, due to a high number of false positives. Specificity was lower in our study than in Chen et al. (Chen Y. et al., 2021); this may be related to our use of TE biopsy results as the reference standard. Therefore, whole-embryo samples should be used as the gold standard in further studies.

Both TE biopsy and NICS have their limitations, that affect the accuracy of the two methods. These may affect the outcomes of assisted pregnancy, such as reduced pregnancy rate, increased abortion rate, etc., Rubio et al. reported that the ongoing pregnancy rates of euploidy embryos screened by TE biopsy and NICS were higher than that of NICS aneuploidy embryos after embryo transplantation, but there was no statistical difference between the two groups (Rubio et al., 2019). In our study, patients who underwent embryo transfer (TE euploid) were divided into two groups: euploid TE/euploid NICS group and euploid TE/aneuploid NICS group, the outcome of assisted pregnancy was retrospectively analyzed between the two groups. The clinical pregnancy rates were 71.2% and 54.5%, and the ongoing rates were 67.3% and 51.5% in the euploid TE/euploid NICS and euploid TE/aneuploid NICS groups, respectively. The clinical and ongoing pregnancy rates in the euploid TE/euploid NICS group were higher than in the euploid TE/aneuploid NICS group, but there was no statistical difference between the two groups. This was consistent with the findings of Rubio et al. The ongoing pregnancy rate in the present study was higher in both groups than that reported by Rubio et al. (52.9%, NCIS euploid; 16.7% NCIS aneuploid) (Rubio et al., 2019). Moreover, there was no significant difference in the abortion rates between the two groups. TE biopsies have the following problems. Embryo mosaicism gives rise to false positives and false negatives in PGT-A because the inner cell mass (ICM) cells, which forms the fetus are not tested by the TE biopsy, so false negatives of TE biopsy are of concern, because their transfer may lead to either no pregnancy or, worse yet, and abnormal fetus (Huang et al., 2019). While the DNA in SCM likely originates from both of the ICM and TE cell lineages (Huang et al., 2019). In our study, the high false positives may have led to insignificant differences in the clinical outcomes between the euploid TE/euploid NICS and euploid TE/aneuploid NICS groups. However, the population is relatively young, with good clinical conditions and a low probability of abortion, which may also have led to an insignificant difference. The sample sizes were small in our study as well as in the study of Rubio et al. (2019); in the latter, only 29 embryos were transferred. Therefore, whether the clinical outcomes can be improved, by selecting embryos that are euploid for both TE biopsies and NICS, needs to be further confirmed by expanding the sample size.

In summary, our results suggest that NICS was similarly effective in assessing both euploid and aneuploid chromosomes in embryos. Therefore, combining the TE biopsy and NICS results may improve the outcomes of assisted pregnancy; however, this needs to be studied further.

## Data Availability

The authors acknowledge that the data presented in this study must be deposited and made publicly available in an acceptable repository, prior to publication. Frontiers cannot accept a manuscript that does not adhere to our open data policies.
