# Correction: Effectiveness of non-invasive chromosomal screening for normal karyotype and chromosomal rearrangements

**DOI:** 10.3389/fgene.2025.1711906

**Published:** 2025-10-27

**Authors:** Bo-Lan Sun, Yong Wang, Sixi Wen, Liang Zhou, Chun-Hui Zhang, Ze-Xuan Wu, Jie Qiao, Qing-Yuan Sun, Ya-Xin Yao, Jing Wang, Zi-Yun Yi, Wei-Ping Qian

**Affiliations:** ^1^ The Reproductive Medicine Center, Peking University Shenzhen Hospital, Shenzhen, Guangdong, China; ^2^ Reproductive Medical Center, Peking University Third Hospital, Beijing, China; ^3^ Fertility Preservation Lab, Reproductive Medicine Center, Guangdong Second Provincial General Hospital, Guangzhou, China; ^4^ Department of Clinical Research, Yikon Genomics Company, Ltd., Suzhou, China

**Keywords:** non-invasive chromosomal screening, assisted reproductive technology, chromosomal ploidy, next-generation sequencing, blastocyst culture medium, clinical outcomes

The funder “Key projects of basic research plan of Shenzhen Science and technology innovation Commission (JCYJ20200109140623124)” was erroneously omitted. The funder has now been added at the beginning of the Funding statement, which now reads as follows:

This study was supported by the Key projects of basic research plan of Shenzhen Science and technology innovation Commission (JCYJ20200109140623124), the Exploration of New Method of Non-invasive Fertility Evaluation and Establishment of National Standard (Grant No. (2018YFC1002104)); The National Natural Science Foundation of China (Grant No. (31530049)); The Research Team of Female Reproductive Health and Fertility Preservation (Grant No. (SZSM201612065)); The Natural Science Foundation of Guangdong Province (Grant No. (2018A030310673)); The Project for Medical Discipline Advancement of Health and Family Planning commission of Shenzhen Municipality (Grant No. (SZXJ2017003)); The National Natural Science Foundation of China (Grant No. (82001541)); and the National Natural Science Foundation of China (Grant No (8200062044)).

The original article has been updated.

